# Soluble Phenolic Composition Tailored by Germination Conditions Accompany Antioxidant and Anti-Inflammatory Properties of Wheat

**DOI:** 10.3390/antiox9050426

**Published:** 2020-05-14

**Authors:** Irene Tomé-Sánchez, Ana Belén Martín-Diana, Elena Peñas, Sara Bautista-Expósito, Juana Frias, Daniel Rico, Lorena González-Maillo, Cristina Martinez-Villaluenga

**Affiliations:** 1 Institute of Food Science, Technology and Nutrition (ICTAN-CSIC), Jose Antonio Novais, 10, 28040 Madrid, Spain; i.tome@ictan.csic.es (I.T.-S.); elenape@ictan.csic.es (E.P.); sara.bautista@ictan.csic.es (S.B.-E.); frias@ictan.csic.es (J.F.); 2 Agricultural Technological Institute of Castile and Leon (ITACyL), Government of Castile and Leon. Ctra. de Burgos Km.119, Finca Zamadueñas, 47071 Valladolid, Spain; mardiaan@itacyl.es (A.B.M.-D.); ricbarda@itacyl.es (D.R.); Lorena.gonzalez@itacyl.es (L.G.-M.)

**Keywords:** antioxidant activity, germination, immunomodulation, nutraceutical value, optimization, phenolic compounds, wheat

## Abstract

Knowledge on the specific variation in the phenolic composition of wheat defined by germination conditions and its relationship with antioxidant and anti-inflammatory properties of sprouts would be useful to improve the functional value of wheat-derived products. Variation in soluble phenolic composition, antioxidant and anti-inflammatory potential of wheat was examined in a range of germination temperature (12–21 °C) and time (1–7 d). Response surface methodology was applied for building lineal and quadratic models to find optimal germination conditions to improve nutraceutical value of wheat sprouts using the desirability (*D*) function. Phenolics were determined by HPLC-DAD-ESI-MS. *In vitro* biochemical methods and lipopolysaccharide stimulated RAW264.7 macrophages were used to determine antiradical and anti-inflammatory activities of wheat sprouts. Accumulation of soluble phenolic acids, flavone *C*-glycosides and lignans in sprouts was positively influenced by germination temperature and time. Increased concentration of individual polyphenols was directly associated with improved ability of sprouts for radical scavenging and reduction of tumor necrosis factor α and interleukin 6 in macrophages. Optimal desirability (*D* = 0.89) for improved nutraceutical value of wheat sprouts was achieved at 21 °C for 7 d. This information would be useful for food industry aiming at producing wheat-based products with better nutritional and healthy properties.

## 1. Introduction

The immune system plays an important role affecting the health and life quality of people [1]. Dysregulation of the immune system leads to the development and complications of chronic diseases [2]. Inflammation is a physiological process for activating the immune system as a defense mechanism against microbial infections, irritant compounds or damaged tissue [3]. Although inflammation in an acute disease state aids in fighting diseases, chronic inflammation at a subclinical level plays a significant role in the initiation and progression of neurodegenerative and cardiovascular diseases, obesity and cancer [4,5]. During chronic inflammation, some cells, mainly macrophages, produce pro-inflammatory cytokines such as interleukins (IL) (IL-1β, IL-6 and IL-8), tumor necrosis factor (TNF)-α, reactive oxygen species (ROS), nitric oxide (NO) and prostaglandins [6]. Since a variety of bioactive dietary components have been shown to affect the immune system, the development of ingredients enriched in immunomodulatory and antioxidant compounds that can counteract impaired immune function and attenuate oxidative stress can be a promising approach for the prevention and management of diseases.

Epidemiological studies have associated whole cereal grain consumption with lower risk of disease, due to their unique composition in health-promoting bioactive components, including fiber and antioxidant phenolic compounds, mainly present in bran and germ fractions [7,8]. Wheat ranks as the second most produced and consumed cereal crop [9]. Whole wheat is an excellent source of antioxidants, such as phenolic compounds, that have been shown to affect the immune system [10]. An anti-inflammatory mechanism known for wheat phenolic compounds is the interaction with cell receptors, activation of signaling cascades and suppression of the transcription of pro-inflammatory cytokines in endothelial cells, monocytes and macrophages [11]. Phenolic compounds in wheat grains are mainly represented by phenolic acids found as free, conjugated and bound forms, with the latter the most abundant [12]. The biological activity of phenolics depends on their bioavailability; therefore, processing methods enhancing the release of phenolics from the food matrix may be a valuable approach to improve the bioefficacy of foods containing phenolics and counteract oxidative stress and impaired immune function.

During the last years, there has been an increased consumer demand for sprouted cereal grain products and a parallel increase in the number of research studies showing the positive impact of germination on nutrients and phytochemicals of grains [13,14]. In the particular case of wheat, a number of studies have shown that germination increases soluble phenolics content and antioxidant activity [14,15,16], although there is still a gap in knowledge on the impact of germination on the immunomodulatory activity of wheat.

A multitude of factors can affect the magnitude of changes naturally occurring during germination. Germination temperature and time have been reported to have an impact on the phenolic composition and antioxidant activity of wheat [14,15,17]. Therefore, optimization of the germination process is a prerequisite to maximize the concentration of bioactive compounds, their bioavailability and, consequently, their health effects. In this sense, the major objective of this study was to identify the germination conditions for an optimal phenolic profile, antioxidant and immunomodulatory activities of wheat sprouts. To this purpose, several experimental conditions were surveyed to study the influence of germination temperature and time on phenolic composition, and the antioxidant and anti-inflammatory potential of whole wheat. Moreover, different multivariate analyses were carried out to optimize processing parameters, to assess the differences of sprouted wheat traits determined by germination and the correlations among individual phenolic content and biofunctional properties.

## 2. Materials and Methods

### 2.1. Chemicals

Lipopolysaccharide (LPS) O55:B5 from *Escherichia coli*, fluorescein, 2,2′-azinobis 3-ethylbenzothiazoline-6-sulfonic acid (ABTS), 2,2′-diazobis-(2-aminodinopropane)-dihydrochloride (AAPH), 2,2-diphenyl-1-picrylhydrazyl (DPPH), 2,4,6-tri(2-pyridyl)-s-triazine, Folin–Ciocalteu reagent, gallic acid, 6-hydroxy-2,5,7,8-tetramethyl-2-carboxylic acid (Trolox) were obtained from Sigma-Aldrich, Co. (Sigma-Aldrich Co., St. Louis, MO, USA). Caffeic acid, ferulic acid, vicenin-2 and podophyllotoxin standards were purchased from Extrasynthese (Lyon, Genay, France). Murine macrophage cell line RAW 264.7 was obtained from the American Type Culture Collection (ATCC, Rockville, MD, USA). High-glucose Dulbecco’s modified Eagle’s medium (DMEM) and penicillin/streptomycin (10,000 U/mL) were purchased from Lonza Group (Lonza, Madrid, Spain). Fetal bovine serum (FBS) was obtained from Hyclone (GE Healthcare, Logan, UT, USA).

### 2.2. Grain Samples

Whole wheat grains (*Triticum aestivum* L., var. Berdún) containing 10% moisture and 11% protein were kindly supplied by Emilio Esteban, S.A. (Emesa S.A., Valladolid, Spain). Wheat was harvested in La Mudarra (Valladolid, Spain) at full ripening stage during the 2018–2019 crop year. They were stored in vacuum-sealed plastic bags at room temperature and darkness. Grain samples were milled (Taurus, Oliana, Spain), sieved using a 0.3 mm screen and stored at −20 °C until analysis.

### 2.3. Germination

Forty grams of whole wheat grains were washed with tap water and hygienized in a solution of 0.5% (*v*/*v*) sodium hypochlorite (ratio 1:6, *w*/*v*) for 30 min. Afterwards, grains were washed with tap water to reach neutral pH and soaked in sterile water (ratio 1:6 *w*/*v*) for 4 h. Soaked wheat grains were spread across a moist filter over a steel grid, which was placed in plastic trays containing sterile tap water. Seeds were covered by moist filter paper and introduced in a germination cabinet (Snijders Scientific, Tilburg, The Netherlands), equipped with a water circulation system that provided a relative humidity >90%. Germination was performed in darkness at different temperatures (12–21 °C) and times (1–7 d). Three replicates of the experimental conditions detailed in Section 2.8 were carried out. Non-germinated whole wheat was included as control. Germinated seeds (sprouts) were freeze-dried, milled (Taurus, Oliana, Spain) and sieved using a 0.3 mm screen. Flours were stored in plastic bags under vacuum at −20 °C until further analysis.

### 2.4. Extraction of Total Soluble Phenolic Compounds

The extraction of total soluble phenolic compounds from control and germinated wheat was conducted according to the method described by Dinelli et al. [18] with some modifications. One gram of flour was extracted with 20 mL of ethanol/water (80:20, *v*/*v*) at 4 °C for 10 min. Subsequently, samples were centrifuged at 2500× *g* at 4 °C for 10 min using a Sorval RC 5B centrifuge (ThermoFisher, Madrid, Spain). The supernatant was collected and the extraction process was repeated once again. The extracts were pooled and evaporated in a rotary evaporator at 175 psi and 40 °C (Rotavapor^®^ R-300, BÜCHI Labortechnik AG, Flawil, Switzerland). Dry extracts were dissolved in 2 mL of methanol/water (80:20, *v*/*v*). Finally, samples were stored at −20 °C until they were used.

### 2.5. Determination of Total Soluble Phenolic Compounds (TSPC)

Total phenolics in hydroalcoholic extracts (free phenolic fraction) was determined according to the Folin–Ciocalteu procedure [19]. Briefly, a volume of 140 µL of the sample extract was mixed with 280 µL of Folin–Ciocalteu reagent previously diluted (1:10, *v*/*v*) and 980 µL of 42.86 mM sodium carbonate. The mixture was shaken and allowed to stand for 100 min in darkness, following centrifugation at 15,000× *g* for 3 min. The absorbance was measured at 760 nm with a microplate reader (Fluostar Omega, Ortenberg, Germany). Calibration curves of gallic acid in a concentration range between 4.5–250 μg/mL showed good linearity (r^2^ < 0.99) and were used for quantification of total phenolic content. Results are expressed as mg of gallic acid equivalents (GAE) per 100 g of sample dry weight (dw).

### 2.6. Characterization of Free Phenolic Profile

Determination of free phenolic profile was carried out by HPLC-DAD-ESI/MS using a method adapted from García-Villalva et al. [20]. Briefly, sample (20 μL) elution was carried out on a Kinetex-F5 column (5 μm, C18, 250 × 4.6 mm; Phenomenex, Macclesfield, UK) at a flow rate of 1 mL/min. Elution gradient of 1% formic acid in water (solvent A) and acetonitrile (solvent B) was performed as follows: from 5% B to 60% B in 37 min, from 60% B to 98% B in 3 min, from 98% B to 5% B in 5 min. HPLC (Agilent Technologies, Santa Clara, CA, USA) was coupled to an ion-trap mass spectrometer equipped with an electrospray ionization (ESI) source (Bruker Daltonics, Billerica, MA, USA). The nebulizer pressure and flow rate of nitrogen were 65.0 psi and 11 L/min, respectively. The heated capillary and voltage were maintained at 350 °C and 3.5 kV, respectively. Mass scan and MS/MS daughter spectra were measured from 100 to 1200 *m/z*. Collision-induced fragmentation experiments were performed in the ion trap using helium as the collision gas, and the collision energy was set at 50%. MS data were acquired in the negative ionization mode. The phenolic compounds were identified according to their maximum absorbance at wavelengths 290 nm and 320 nm, molecular mass and fragmentation pattern. Four calibration curves were prepared using four commercial standards of caffeic acid, ferulic acid, vitexin and vicenin-2 standards showing good linearity over the study range (see Table S1). Data were expressed as mg/100 g dw.

### 2.7. Determination of Antioxidant Activity

#### 2.7.1. Determination of Oxygen Radical Absorbance Capacity (ORAC)

The antioxidant activity was determined by the ORAC method as described previously [21]. Briefly, the reaction was performed at 37 °C in 75 mM phosphate buffer at pH 7.4. The reaction mixture (200 μL) contained 180 μL of 70 nM fluorescein, 90 μL of 12 mM AAPH and 30 μL of diluted sample or the standard Trolox at concentrations ranging from 8 to 160 μM. Reaction mixtures were placed in a black 96-well plate (Fisher Scientific, Waltham, MA, USA) and the fluorescence was read in a Synergy HT microplate reader (BioTek Instruments, Winooski, VT, USA) every minute at excitation and emission wavelengths of 485 and 520 nm, respectively. The equipment was controlled by Gen5™ software, version 1.1 (BioTek Instruments, Winooski, VT, USA). Quantification of ORAC was made according to the linear calibration curves of standard Trolox (r^2^ > 0.99). Results were expressed as µmol Trolox equivalents (TE) per 100 g dw.

#### 2.7.2. Determination of ABTS Radical Cation Scavenging Activity

The ABTS assay was carried out following a method described previously [22]. A stock ABTS solution was prepared by mixing a 7 mM aqueous ABTS solution with 2.45 mM K_2_O_8_S_2_ in a 1:1 (*v*/*v*) ratio. Before the assay, the stock ABTS solution was diluted with phosphate buffer (75 mM, pH 7.4) to obtain a working solution with an absorbance value of 0.70 ± 0.02 at 734 nm. A volume of 20 µL of diluted samples was mixed with 200 µL of ABTS working solution in a 96-well microplate. The decay in absorbance at 730 nm was monitored over 30 min in a microplate reader (Spectrostar Omega, Ortenberg, Germany). Values were expressed as µmol TE/100 g dw using a Trolox calibration curve as external standard in a concentration range from 7.5 to 240 µM.

#### 2.7.3. Determination of Ferric Reducing Antioxidant Power (FRAP)

The method was carried out as described by Benzie and Strain [23] with slight modifications. The FRAP reagent was prepared by mixing 38 mM sodium acetate anhydrous in distilled water pH 3.6, 20 mM Fe(III) Cl_3_ in distilled water and 10 mM 2,4,6-tri(2-pyridyl)-s-triazine in 40 mM HCl in proportions of 10:1:1. This reagent was freshly prepared before each experiment. Reaction mixtures were kept at 37 °C for 40 min in heating blocks, covered with tin foil. The absorbance of the supernatant was monitored at 593 nm using a microplate reader (BioTek Instruments, Winnoski, VT, USA). Results were expressed as mmol Fe^2+^/g dw.

#### 2.7.4. Determination of DPPH Radical Scavenging Activity

The DPPH assay was performed as previously reported [24]. Briefly, a working DPPH methanolic solution (100 µM) was prepared. Aliquots of 125 µL of DPPH^·^ working solution were placed in a 96-well microplates and mixed with 25 µL of sample extracts and 100 µL of bidistilled water. The kinetic of absorbance at 515 nm was recorded after 30 min using a microplate reader (Spectrostar Omega, Ortenberg, Germany). Values were calculated from a standard calibration curve of Trolox (7.5–240 μM) and expressed as µmol TE/100 g dw.

### 2.8. Determination of Anti-inflammatory Activity

The murine macrophage cell line RAW 264.7 was maintained and cultivated as recommended by the American Type Culture Collection. Cells were seeded at a density of 2 × 10^5^ cells/well in 24-well plates. Cells were pre-exposed for 1 h to a sample extract concentration equivalent to 20 mg of flour per mL of growth media. Afterwards, cells were stimulated using 1 µg/mL of LPS for 23 h. Untreated cells were included as a negative control of inflammation and LPS-treated cells without sample extracts were the inflammatory model of reference. After 24 h, supernatant was collected for quantification of TNF-α and IL-6 by enzyme-linked immunosorbent assay kits (Diaclone, Besaçon, France). The absorbance was read at 450 nm using a Synergy HT microplate reader (Biotek Instruments, Winooski, VT, USA). External calibration curves of TNF-α and IL-6 in a linearity range between 0.5–500 pg/mL showed good correlation with absorbance values (r^2^ > 0.99). All extracts were analyzed in duplicate. Results were expressed as percentage inhibition relative to LPS-treated cells without sample extracts. Cell viability was determined in 96-well plates seeded at a density of 5 × 10^4^ cells/well using the Cell Titer 96^®^ AQueous One Solution Proliferation Assay kit (Promega Biotech Ibérica, Madrid, Spain). Cell viability was expressed as percentage relative to untreated cells.

### 2.9. Experimental Design and Data Modeling by Response Surface Methodology

The interrelation between germination temperature/time and phenolic composition and antioxidant and anti-inflammatory activities was determined using the response surface methodology (RSM). RSM is based on the application of multivariate design of experiments (DOE) followed by mathematical modeling and multiple response optimization using desirability (*D*) function. A face-centered rotational composite experimental design built with two-level (–1 and +1) factorial design points, axial and center points was employed. Germination temperature and time were selected as independent factors. The range between factor levels was chosen on the basis of the literature information: from 12 to 21 °C for temperature and from 1 to 7 d for germination time [25]. Soluble phenolic composition, antioxidant and anti-inflammatory parameters were chosen as response variables to be subjected to the optimization procedure because they can provide the necessary information in the evaluation of the nutraceutical value of wheat sprouts and their potential to attenuate inflammatory response and oxidative stress. Experiments were performed in a randomized order to assure the independence of the results.

The experimental data set were used to statistically model the relationship between response variables and experimental factors via multiple regression fitted with the classical least squares (LS) method. For RSM modeling, a mathematical model was built for each response fitting a second order polynomial function. The response (Y) was described by the following regression model (Equation (1)):

(1) $$Y=\beta_{0}\;+\sum_{i=1}^{2}\beta_{i}\;X_{i}+\sum_{i=1}^{2}\beta_{\mathrm{ii}}\;{X_{i}}^{2}+\sum_{i=1}^{2}\beta_{\mathrm{ij}}\;X_{i}\;X_{j}$$

where β_0_ is a constant coefficient, β_i,_ β_ii_, and β_ij_ are the linear, quadratic, and the interaction coefficients, respectively. X_i_ and X_j_ are the germination factors temperature and time, respectively. The significance of the coefficients was investigated through ANOVA, using the Statistica v.7.0 software (Statsoft, Tulsa, OK, USA).

An ANOVA test was applied to determine the significance of the second order model. The latter was considered satisfactory when the regression was significant, and a non-significant lack of fit was obtained for the selected confidence level (α = 0.05). In addition, the coefficient of determination (*R*^2^) and the adjusted coefficient of determination (*R-adj*^2^) were evaluated to confirm that variation of the data was mainly explained by the model. Three-dimensional response surface plots were obtained to illustrate the effects of the independent factors on the response variables. Multiresponse optimization was performed using the Desirability Function in Statistica v.7.0. The following desirability criteria were selected: maximize the concentration of soluble phenolic compounds, antioxidant and anti-inflammatory properties in wheat sprouts.

### 2.10. Statistical Analysis

Data were expressed as the mean ± standard deviation. One-way analysis of variance (ANOVA) and *post hoc* Duncan’s test was performed to identify differences between mean values using Statgraphics Centurion XVIII (Statgraphics Technologies, The Plains, VA, USA). Multivariate analysis of variance (MANOVA) test was used to identify significant effects of germination factors (temperature and time) on response variables. Pearson correlation coefficients and principal component analysis (PCA) were performed on centered and standardized data to elucidate the relationships among variables of the phenolic profile and bioactivity of samples.

## 3. Results

### 3.1. Effect of Germination Conditions on Total and Individual Soluble Phenolic Content of Wheat

The soluble polyphenol content of whole wheat grain (control) and sprouts obtained at different experimental conditions is presented in Table 1. The TSPC of whole wheat was 17.9 mg GAE/100 g dw, a lower concentration compared to the one observed for wheat sprouts. Germination induced a variable increase in the TSPC depending on germination conditions. The lowest value (19.2 mg GAE/100 g dw) was observed in wheat germinated at 16 °C for 1 d and the highest (66.5 mg GAE/100 g) when germination took place at 16 °C for 7 d.

Tentative identifications of soluble phenolic compounds in extracts from wheat grains and sprouts were obtained based on accurate mass measurements and literature comparison. A total of fourteen phenolic compounds were identified in wheat samples belonging to the phenolic acids, flavonoids and lignans classes (see Supplementary Material, Figure S1 and Table S2). Phenolic acids were the most abundant phenolic class detected in all wheat samples accounting for 63.4% of total phenolic acids (Table 1). Caffeic acid *O*-hexoside (CAH) and, dihydroferulic acid isomer 1 (DFA i1) and ferulic acid (FA) were observed in most of the samples, while DFA i2 and feruloylquinic acid (FQA) were exclusively detected in sprouts (Table 1). Flavones were the most representative group of flavonoids in the free wheat extracts accounting for 36.6% of total phenolic compounds in non-germinated grain. Seven *C*-glycosilated apigenins and methyl isoorientin-2-*O*-rhamnoside (MIR) were identified in wheat grains and sprouts. Apigenin-6-*C*-galactosyl-8-*C*-glucosyl-*O*-glucuropyranoside (AGGG), MIR, isovitexin (IVT) and vitexin (VT) were present only in wheat sprouts (Table 1). Similarly, lignans such as 1-acetoxy pinoresinol (1-AP) were exclusively found in wheat sprouts.

Comparing the phenolic profile of sprouts, significant differences were observed among experimental groups for the content of the fourteen individual phenolic compounds analyzed. For the total sum of individual polyphenols concentration, wheat germinated at 16 °C for 7 days and 21 °C for 4 days were able to accumulate the highest amounts of free phenolics (150 and 157 mg/100 g dw, respectively). In these samples, phenolic fraction was composed of 57–58% of phenolic acids, 38–39% of flavonoids and 3–5% of lignans.

In order to highlight the contribution of germination temperature and time to the variability of TSPC and content of individual phenolic compounds, MANOVA analysis was performed. Mean of squares and significance levels for each independent factor are shown in Table 1. A significant effect of temperature and time was observed for most of the parameters studied, with the exception of FA whose variability only depended on germination time. Comparing the effects of both factors, germination time had a prominent effect on the amounts of TSPC and individual phenolic compounds of wheat sprouts, except for 1-AP and AGGG that were mainly influenced by temperature. 

### 3.2. Effect of Germination Conditions on Antioxidant Activity of Wheat

For a complete evaluation of the total antioxidant activity of wheat samples, multiple assays based on different antiradical mechanisms were performed: ORAC (HAT assay), ABTS, DPPH and FRAP (SET assays). Antioxidant activity of wheat grains and sprouts is presented in Table 2. Germination conditions produced a significant variation in the antioxidant activity of wheat. Production of wheat sprouts at 12–16 °C for short times (1–2 d) showed the lowest ORAC, ABTS, FRAP and DPPH values, where the antioxidant activity of these sprouted samples was non-significantly different from control. In contrast, the highest values for ORAC (2058.3–2352.9 µmol TE/100 g dw), ABTS (2862.5–2985.9 µmol TE/100 g dw), FRAP (0.12–0.16 mmol Fe^2+^/g dw) and DPPH (320.7–371.8 µmol TE/100 g dw) assays were observed after wheat germination at 16–21 °C for longer times (6–7 d). Antioxidant activity determined by the four in vitro assays was more influenced by germination time than temperature (MANOVA, *p* ≤ 0.05).

### 3.3. Effect of Germination Conditions on Anti-Inflammatory Activity of Wheat

Anti-inflammatory activity of soluble extracts obtained from wheat grain (control) and wheat sprouts were studied in the well-known LPS-induced RAW264.7 macrophage cell model. Firstly, cell viability was measured after treatment with wheat extracts at a concentration equivalent to 20 mg flour/mL for 24 h. Non-significant (*p* ≤ 0.05) differences in cell viability between treated and non-treated cells confirmed that wheat extracts did not exert a cytotoxic effect (data not shown).

To evaluate the inhibitory effects of wheat sample extracts on inflammation, RAW264.7 cells were pre-exposed for 1 h to extracts at a concentration equivalent of 20 mg of sprouted wheat flour per mL of cell media, before stimulation with LPS (1 μg/mL). Extract concentration was chosen based on preliminary studies showing an effective reduction on pro-inflammatory cytokine release in the same model of inflammation (data not shown). LPS stimulation of RAW264.7 cells increased levels of TNF-α from 1869.7 to 5159.7 pg/mL of growth media and IL-6 from non-detected to 222.8 pg/mL of growth media (data not shown). Extract from wheat grain (control) inhibited the LPS-induced production of TNF-α and IL-6 by about 60% (Table 2).

Anti-inflammatory activity of wheat sprouts showed a significant variation that was highly dependent on germination time and temperature (Table 2). In this way, the lowest inhibition for both TNF-α and IL-6 levels was observed at 12 °C for 2 d (67.6 and 42.9% inhibition, respectively). In contrast, the highest values corresponded to extracts from wheat sprouts at 16 °C for 4 d (76.8% and 74.8% inhibition of TNF-α and IL-6 levels, respectively). MANOVA indicated that germination time showed a prominent effect on the ability of extracts to inhibit pro-inflammatory cytokines (Table 2). Inhibitory effects of extracts on IL-6 production in LPS-stimulated macrophages were exclusively affected by germination time.

### 3.4. Modeling Germination Conditions

Response surface models were built to describe the effect of processing parameters (temperature and time) on wheat phenolic composition and bioactivity after germination. Models with best fitting results (*p*-values = 0.0400–0.0001 and non-significant lack of fit) are visualized in Table 3. Only regression coefficients with probability levels *p* < 0.05 were included in the models. The coefficient of determination (*R*^2^) and the adjusted coefficient of determination (*R*^2^-*adj*) represent the percentage of variance explained by the model. RSM models were considered suitable for explaining experimental data when both *R*^2^ and *R*^2^-*adj* were ≥0.75 [26]. Models generated for AAH, APH (i2), DFA (i1 and i2), FA, IVT, MIR and V-2, FRAP and IL-6 showed *R*^2^ < 0.75, implying that the polynomial models explained less than 75% of the variability of experimental data, and therefore they were omitted in the multiple response optimization.

The ANOVA tests applied to response variables indicated that TSPC and DPPH had a better fit to a linear model (Table 3). Most phenolic compounds, ORAC, ABTS and TNF-α followed a second polynomial model, while FQA variability was explained exclusively by the interaction of germination time and temperature.

**Figure 1:**
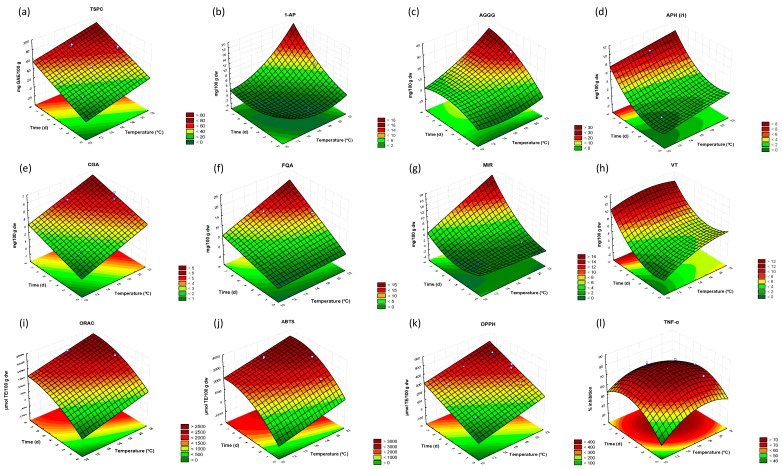
Response Surface 3D contour plots for combined effect of germination time and temperature on (**a**) total soluble phenolic content (TSPC), (**b**) 1-acetoxypinoresinol (1-AP), (**c**) apigenin-6-*C*-galactosyl-8-*C*-glucosyl-*O*-glucuropyranoside (AGGG), (**d**) apigenin-6/8-*C*-pentoside-8/6-*C*-hexoside (APH i1), (**e**) caffeic acid *O*-hesoxide (CAH), (**f**) feruloylquinic acid (FQA), (**g**) methyl isoorientin-2-*O*-rhamnoside (MIR), (**h**) vitexin (VT), (**i**) oxygen radical absorbance capacity (ORAC), (**j**) 2’-azino-bis(3-ethylbenzothiazoline-6-sulfonic acid) (ABTS); (**k**) 2,2-diphenyl-1-picryl-hydrazyl-hydrate (DPPH), (**l**) tumor necrosis factor -α inhibition (TNF-α). Blue dots represent the data of the experimental groups of samples.

The relationship between factors and response variables as well as the optimal germination conditions for a maximum response is represented in three-dimensional surface contour plots (Figure 1). Temperature exerted positive linear and/or quadratic effects on TSPC, 1-AP, APH (i1), AGGG, CAH, VT, ORAC, ABTS, DPPH and TNF-α. Similarly, a positive linear and/or quadratic effect of time was described for all response values, indicating that total and individual soluble phenolic compounds, antioxidant and anti-inflammatory parameters increased with germination time (Figure 1). Negative linear or quadratic effects of temperature were observed for 1-AP, AGGG, VT and TNF-α, while negative linear or quadratic effects of time were found in 1-AP, APH (i1), AGGG, MIR, ORAC, ABTS and TNF-α. This means that in some areas of the response surface plots, response values decreased with increasing levels of the processing factors. Finally, the interaction of time and temperature showed significant effects on 1-AP, AGGG, FQA, MIR, VT and TNF-α.

A comparison of the regression coefficients of linear, quadratic and interaction terms in the models (Table 3) indicated that most of the response variables were mainly influenced by linear and/or quadratic effects of germination time. Some exceptions to this observation were AGGG and VT in which the linear effect of temperature was predominant, while FQA was affected exclusively by the interaction of temperature and time.

### 3.5. Multiple Response Optimization of Germination Parameters and Validation of Predictive Models

The desirability (*D*) function was applied to find germination conditions that simultaneously optimize response variables with statistically validated models. The global desirability function was plotted, reaching a level of *D* = 0.8912 (Table 4) for the following criteria: maximum total and individual phenolic content and maximum efficiency for antioxidant and anti-inflammatory activities. The experimental conditions providing the highest efficiency to maximize the nutraceutical value of sprouted wheat were 21 °C and 7 d (Table 4). Under these optimal conditions wheat sprouts were predicted to contain 74.4 mg/100 g of TSPC, 7.9 mg/100 g of 1-AP, 7.3 mg/100 g of APH (i1), 20.7 mg/100 g of AGGG, 5.9 mg/100 g of CAH, 14.5 mg/100 g of FQA, 9.4 mg/100 g of MIR and 10.6 mg/100 g of VT. Antioxidant activity was predicted to be 2490.3, 3384.4, and 430.2 μmol TE/100 g as determined by ORAC, ABTS and DPPH assays, respectively. Finally, a concentration of extract equivalent to 20 mg/mL of sprouted wheat flour was predicted to inhibit 71.7% of the release of TNF-α.

The desirability model was validated by comparing predicted and experimental data obtained for a combination of factors levels within the optimal *D* region that included germination temperatures between 16–22 °C and times between 6–8 days (see Supplementary Material, Figure S2). Experimental values were not statistically different (one-way ANOVA, *p* ≥ 0.05) compared to those predicted at 20 °C for 6 d (Table 4), results that validated the model optimization.

### 3.6. Differences in Phenolic Composition and Functional Properties of Wheat Sprouts as Function of Germination Conditions

**Figure 2:**
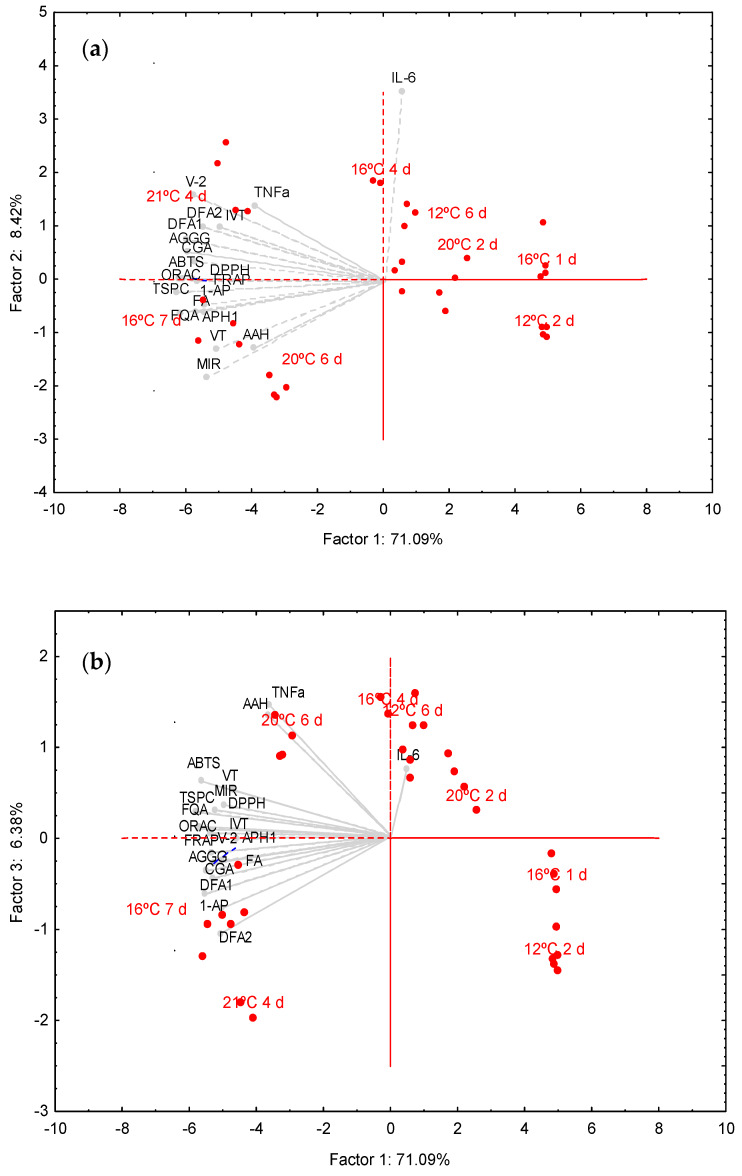
Bi-plots of PCA. Factor 1 vs. Factor 2 (**a**) and Factor 1 vs. Factor 3 (**b**) separates the germination experiments based on phenolic composition and bioactivity (antioxidant and anti-inflammatory properties) of wheat. Abbreviations: 2,2’-azino-bis(3-ethylbenzothiazoline-6-sulfonic acid) (ABTS); 1-acetoxypinoresinol (1-AP); apigenin-6-*C*-arabinoside-8-C-hexoside (AAH); apigenin-6-*C*-galactosyl-8-*C*-glucosyl-*O*-glucuropyranoside (AGGG); apigenin-6/8-*C*-pentoside-8/6-*C*-hexoside (APH); caffeic acid *O*-hexoside (CAH); dihydroferulic acid (DFA); 2,2-diphenyl-1-picryl-hydrazyl-hydrate (DPPH); ferric reducing antioxidant power (FRAP); feruloylquinic acid (FQA); interleukin (IL); isomer (i); isovitexin (IVT); methyl isoorientin-2-*O*-rhamnoside (MIR); oxygen radical absorbance capacity (ORAC); total soluble phenolic compounds (TSPC); tumor necrosis factor (TNF); vicenin-2 (V-2); vitexin (VT).

PCA was carried out to obtain a detailed description of differences in phenolic composition and bioactivity among wheat sprouts determined by different germination conditions (Figure 2).

The outcome of the PCA of the eight experimental conditions of wheat germination is represented in two dimensions, where the *x*-axis plots the values of first principal component (Factor 1) and the *y*-axis plots the values of the second (Factor 2, Figure 2a) and third (Factor 3, Figure 2b) principal components. The cumulative percentage of variance explained by the first three principal components was 85.9% of which Factor 1, 2 and 3 explained 71.1%, 8.4%, 6.4% of the total variance. Points represent wheat treatments, while the vectors are related to phenolic profile, antioxidant and anti-inflammatory parameters. Factor 1 showed high negative loadings (>−0.8) for TSPC, individual phenolic compounds (except for AAH), all antioxidant parameters and inhibition of TNF-α; therefore, the negative branch of this component was associated with higher phenolic content, antioxidant activity and ability to reduce TNF-α. Factor 2 and 3 had high positive loadings for IL-6 and AAH, respectively. The PCA allowed a clear clustering between wheat sprouts obtained at different germination conditions. Wheat germinated at 12 °C for 2 d and 16 °C for 1 d were clustered in the right part of the bi-plots showing low total and individual phenolic content, antioxidant and anti-inflammatory activities (Figure 2a,b). A separated group of wheat sprouts obtained at 12 °C for 6 d, 20 °C for 2 d and 16 °C for 4 d were located near the center-up part of the bi-plots, which was indicative for intermediate phenolic content, antioxidant activity and inhibition of TNF-α and at the same time, strong inhibitory effects of IL-6 (Figure 2a,b). Finally, wheat sprouts germinated at 20 °C for 6 d, 16 °C for 7 d and 21 °C for 4 d were grouped in the left side of bi-plots (Figure 2a,b), due to their high phenolic content and antioxidant activity. Within this group of samples, wheat germinated at 21 °C for 4 d was separated in the upper part of Figure 2a, which was indicative of a higher inhibition of IL-6. Similarly, wheat sprouts obtained at 20 °C for 6 d were separated in the upper part of Figure 2b due to its higher content of AAH.

The relationships between phenolic composition and bioactivity of wheat samples was examined across the different experimental groups by correlation analysis (Table 5).

While some parameters appeared unrelated across the correlation matrix, others showed significant correlations. TSPC was positively correlated with ORAC, ABTS, FRAP, DPPH and TNF-α, indicating that the increase in phenolic compounds is related to greater antioxidant and anti-inflammatory activities. Although most individual phenolic compounds were positively associated with all antioxidant parameters, other compounds such as AAH, IVT and VT were non-significantly correlated with ORAC. Of note, APH (i1), AGGG, DFA (i1), FQA and V-2 showed the strongest correlations with antioxidant parameters. As regards to anti-inflammatory parameters, inhibition of TNF-α showed a positive relationship with most phenolic compounds (except for 1-AP) and, in particular, V-2 showed the strongest correlation. Finally, no significant correlation was observed between phenolic content and inhibition of IL-6. These results suggest that antioxidant and anti-inflammatory effects of wheat sprouts are also largely associated with soluble phenolic composition and, therefore, optimizing phenolic composition by germination is crucial in the enhancement of functional properties of grains.

## 4. Discussion

It is well established that oxidative stress and a chronic inflammatory state are the underlying causes in the onset and development of many chronic degenerative diseases [4,5]. Epidemiological research has proven that diet plays a pivotal role in the prevention/counteraction of oxidative and inflammatory damage [27]. In this context, using raw materials with high nutraceutical value and the optimization of industrial operations for more efficient functional properties offer a great opportunity for the development of healthy foods. The objective of this study was to produce a sprouted wheat-derived flour with superior antioxidant and anti-inflammatory properties by means of optimization of the grain germination process.

Wheat was selected for this study, firstly, due to the existence of epidemiological evidence associating wholegrain consumption with beneficial health effects [7]. Secondly, being a staple food, wheat plays an important role in human nutrition, providing not only essential nutrients (i.e., protein, starch, lipids) but also antioxidants and anti-inflammatory compounds that confer unique health promoting properties to wheat-derived products [12]. In particular, phenolic compounds are the most representative class of phytochemicals in wheat grains with the ability to counteract oxidative stress and inflammation [28]. The phytochemical profile of wheat is highly dependent of genotype, environmental and growing conditions [29,30]. A comparison of *Triticum aestivum* var. Berdún with previous studies focused on the characterization of old and modern common wheat genotypes indicated that the wheat variety used in this study had similar total soluble phenolic content and profile (in descending order of abundance FA, CAH and apigenin *C*-glycosides; Table 1) to the others reported previously [18,30].

The majority of the phenolic compounds in wheat are bound to cell wall polysaccharides, having very low bioavailability that hinder their ability to exert physiological effects *in vivo*. In the present study, germination was used as a sustainable processing method to boost the accumulation of soluble phenolic forms in wheat grains. Natural activation of endogenous cinammoyl and feruloyl esterases and the biosynthesis of phenolics via the shikimate and phenylpropanoid pathways are the main mechanisms explaining the increase of soluble phenolic content in sprouts [31]. Germination may also impact grain microstructure by increasing the natural porosity of cell walls through activation of endoxylanases, enzymes involved in the disintegration of cell wall arabinoxylans [32]. These biochemical changes have been associated with better extractability and bioaccessibility of phenolic compounds [33].

As a natural biological process, physical and biochemical events occurring during seed germination are influenced by genotype, environmental factors such as temperature and the stage of germination development [13]. Therefore, knowledge on the specific variation in the phenolic profile of wheat as affected by germination conditions, and its relationship with the antioxidant and anti-inflammatory properties of sprouts, could be useful to improve their functional value and produce superior wheat-derived products. In order to investigate the variation in wheat phenolic composition, antioxidant and anti-inflammatory activities induced by germination conditions, a wide range of temperature (12–21 °C) and time (1 to 7 d) was studied. Although wheat germination and seedling growth take place in a wider range of temperature (10–30 °C), experiments were performed between 12 and 21 °C considering previous research showing significant losses in dry matter (up to 15%) at temperatures above 20 °C in wheat sprouted for 3 d [25]. Growth and respiration processes were the major factors responsible for these changes in dry matter. Moreover, there is a large body of evidence showing that warm temperatures (22–24 °C) promote rapid microbial growth during seed sprouting and the risk of pathogenic contamination [34]. This was another aspect that determined our experimental design and its future applicability to the food industry.

### 4.1. Variation in Total and Individual Phenolic Content during Wheat Germination

Our results showed up to 3.7-fold increase for TSPC in sprouts of wheat variety Berdún, a value in the upper level of the range reported in the literature (1.2–3.6 fold increase) for cereals sprouted at 15 to 28 °C for 2 to 6 days [35]. New phenolic acids (DFA isomers, FQA), flavone C-glycosides (IVT, MIR, V-2 and VT) and lignans (1-AP) accumulated in wheat sprouts although a variation in the individual content of phenolic compounds was observed among experimental conditions (Table 1). MANOVA results confirmed that temperature, and to a higher extent time, were the factors determining the changes in phenolic composition, with the exception of FA, which was exclusively affected by germination time (Table 1). Complementary results were obtained from RSM, indicating that phenolic variation during wheat germination depended on linear, quadratic and/or interaction effects of temperature and time (Table 3). Overall evaluation of results from RSM suggested that temperature and time positively influenced the accumulation of phenolic acids, flavonoids and lignans (Figure 1). This means that higher germination temperature and time bring about a higher accumulation of all phenolic classes in wheat sprouts. To the best of our knowledge, this is the first work showing the effect of germination temperature and time on wheat flavone *C*-glycosides and lignans.

In accordance with our results, existing evidence has demonstrated the beneficial effects of germination for long periods on the total content of phenolic acids of wheat and other cereals [36,37]. Kim et al. [14] observed that soluble phenolic acids of *T. aestivum* var. Keumkang including gallic, 4-hydroxybenzoic, vanillic, caffeic, syringic, ferulic and *p*-coumaric acids increase time-dependently (from 1 to 4 d) during germination at 25 °C. Similarly, Chen et al. [17] reported a noticeable increase of free *p*-hydroxybenzoic, syringic, *p*-coumaric, ferulic and sinapic acids as germination at 25 °C of three Chinese varieties of wheat progressed from 1 to 4 d. In these previous studies, the free phenolic acid composition of wheat sprouts in order of abundance was syringic > sinapic > ferulic > coumaric > caffeic. In wheat sprouts from variety Berdún, FA and DFA were the most abundant phenolic acids followed by lower quantities of FQA and CAH. Genotype effects and their interaction with germination factors (time and temperature) could explain these differences on phenolic acid composition of sprouts from different varieties of *T. aestivum* [17,38,39]. Recent research on rice germination provides some insights on the molecular basis of accumulation of soluble phenolic compounds as function of germination time [36]. Higher gene expression of phenolic biosynthetic enzymes (four phenylammonia-lyases, chalcone synthase, cinnamoyl-CoA reductase, 4-coumarate-CoA ligase, two cinnamyl alcohol dehydrogenases) at sprouting times of 120 h, as compared to 36 h, promoted higher phenolics accumulation in Chinese wild rice.

RSM results have proven that total and individual phenolic accumulation during germination is influenced by linear and/or quadratic effects of temperature in the range studied (12 to 21 °C; Table 3 and Figure 1a–h). Consistent with our results, Dziki et al. [40] demonstrated that wheat germination at 15–25 °C for 2 to 8 d fits a quadratic model reflecting an increase of TSPC up to 20 °C, after which it decreases to reach lower values at 25 °C. As regards phenolic composition, Swieca and Dziki [41] reported a higher accumulation of syringic, vanillic, *p*-hydroxybenzoic and ferulic acids in wheat germinated at 20 °C for 2 to 4 d than in the counterparts germinated at 25 °C.

### 4.2. Variation in Antioxidant Activity during Wheat Germination

Wheat variety Berdún exhibited values within the range of the ones reported in earlier studies (1786 µmol TE/100 g dw for ORAC, 359–1944 µmol TE/100 g dw for ABTS, 0.02–3.14 mmol Fe^2+^/g dw for FRAP and 116–910 µmol TE/100 g dw for DPPH) [14,17,29,30]. Generally, wheat sprouts showed higher ability to scavenge radical species than non-germinated grains (up to 2.6, 2.8, 1.5, 1.7-fold increase for ORAC, ABTS, FRAP and DPPH, respectively) (Table 2), although significant variations among germination conditions were observed. Time, and secondly, temperature were determinants for the observed variation in antioxidant activity of sprouts (MANOVA, Table 2). RSM results indicated that the variation in antioxidant activity fitted quadratic regression models in which scavenging activity declined initially at early germination, after which it increased with higher temperature and time (Table 3, Figure 1i–k). Similar observations have been reported by our research group in germinated barley at 12–20 °C for 0.8–6 d [36], germinated rice at 28–34 °C for 2–4 d [42] and germinated purple corn at 12–28 °C for 0.5–3 d [43].

### 4.3. Variation in Anti-inflammatory Activity during Wheat Germination

Given the importance of macrophages on innate immune function, the anti-inflammatory activity of wheat variety Berdún was evaluated in a cellular model of proinflammatory state induced in macrophages by LPS. Results indicated that soluble extracts equivalent to 20 mg of wheat flour had the ability to strongly reduce the release of proinflammatory cytokines (TNF-α and IL-6) into cell media, highlighting the potential of variety Berdún to modulate the inflammatory response (Table 2). These results confirm previous evidence showing the reduction of the inflammatory response of a phenolic extract equivalent to 10 mg of biofortified wheat bread in LPS-induced human umbilical endothelial cells [11]. Wheat sprouts of variety Berdún also showed the ability to reduce the release of proinflammatory cytokines *in vitro*, which is consistent with the literature. Ki et al. [44] reported that oral administration of ethanolic extracts from sprouted wheat (200 mg/kg) attenuated serum levels of TNF-α, IL-1β, IL-6, cyclooxygenase-2, and inducible nitric oxide synthase in a murine model of colorectal carcinogenesis.

Variation in the anti-inflammatory activity of wheat sprouts was associated with germination temperature and mostly time (Table 2). Inhibition of TNF-α by sprouted wheat extracts fitted to a quadratic regression model in which the inhibitory effect on TNF-α increased with temperature and time up to 20 °C and 5 d and, subsequently, it decreased (Table 3, Figure 1l). To the best of our knowledge, this is the first study investigating the effects of time and temperature on the anti-inflammatory potential of sprouted wheat.

### 4.4. Explaining Differences in Phenolic Profile and Bioactivity of Sprouted Wheat as a Function of Germination Conditions

In this study, PCA was performed to identify how differences in germination conditions relate to phenolic acid composition, antioxidant and anti-inflammatory properties of wheat. Sprouts with lower phenolic content and bioactivity were the ones obtained at 12 °C for 2 d and 16 °C for 1 d (Figure 2). In contrast, sprouts with higher nutraceutical value were those produced at 20 °C for 6 d, 16 °C for 7 d and 21 °C for 4 d. Based on these results, is seems likely that differential accumulation of phenolic compounds determined by germination conditions contribute directly to differences in antioxidant and anti-inflammatory potential of sprouts.

Correlation analysis confirmed the relationships among variables derived from PCA (Table 5). At the same time, APH (i1), AGGG, DFA (i1), FQA and V-2 were identified as the main contributors to the variation in antioxidant activity while V-2 was the main determinant of the inhibition of TNF-α. Finally, a non-significant correlation between IL-6 inhibition and phenolic content indicated that this biological effect could rely on other wheat compounds. Previous research focused on sprouted brown rice provide some evidence to this. Proteomic and genomic analysis revealed a link between increased antioxidant activity, phenolics accumulation of sprouted brown rice and molecular pathways influencing upregulation of proteins involved in the biosynthesis of phenolics and protection of oxidative stress [37,44]. Consistent with rice studies, other researchers have provided evidence on the association of accumulation of phenolics and antioxidant activity during sprouting of wheat grains [16,17,38].

Taking all together, our results suggest that the appropriate germination of wheat may be a method to improve health-related benefits as a result of an increased content of important metabolites. A multiple response optimization approach identified that sprouting temperature at 21 °C for 7 d was appropriate to achieve the optimal phenolic composition, antioxidant and anti-inflammatory potential in the wheat variety Berdún. As compared to traditional whole-wheat flour, optimal germination conditions were predicted to provide a sprouted wheat flour with 4.2-fold higher soluble phenolic content (including a wider diversity of metabolites), between 1.9–3.2-fold higher capacity to scavenge radicals and up to 1.5-fold higher capacity to modulate cytokine secretion by macrophages in a model of acute inflammation.

## 5. Conclusions

From this study, it is clear that temperature and time are determinant factors during wheat germination that contribute to differential phenolic profile, antioxidant and anti-inflammatory activities. In particular, we provide evidence that higher temperature (up to 21 °C) and time (up to 7 d) during wheat germination is manifested in the accumulation of soluble phenolic acids, flavone *C*-glycosides and lignans and, consequently, the enhancement of its potential to attenuate oxidative stress and inflammatory response. Wheat germination at 21 °C for 7 d is highly recommended for an optimal composition in soluble phenolics and bioactive properties. This information would be useful for the food industry aiming at producing wheat-based products of higher value in terms of nutritional and healthy properties.

## Figures and Tables

**Table 1:** Total soluble and individual phenolic content of whole-wheat grain (control) and sprouts obtained at different temperatures and times.

T (°C)	t (Days)	TSPC	1-AP	AAH (i1)	APH (i1)	APH (i2)	AGGG	CAH	DFA (i1)	DFA (i2)	FA	FQA	IVT	MIR	V-2	VT
Control		17.91 ± 0.54 ^a^	n.d.	0.75 ± 0.05 ^a^	0.69 ± 0.16 ^ab^	0.23 ± 0.01 ^a^	n.d.	1.48 ± 0.47 ^a^	0.65 ± 0.02 ^a^	n.d.	2.54 ± 0.11 ^a^	n.d.	n.d.	n.d.	n.d.	n.d.
12	2	22.12 ± 1.82 ^b^	n.d.	n.d.	n.d.	n.d.	n.d.	1.97 ± 0.05 ^ab^	n.d.	n.d.	11.30 ± 2.08 ^bc^	n.d.	n.d.	n.d.	n.d.	1.26 ± 0.00 ^a^
12	6	43.00 ± 3.33 ^c^	n.d.	6.53 ± 0.24 ^c^	2.24 ± 0.16 ^bcd^	1.42 ± 0.20 ^b^	5.29 ± 0.92 ^b^	2.83 ± 0.01 ^ab^	8.12 ± 0.70 ^c^	5.84 ± 1.27 ^a^	12.78 ± 1.64 ^bcd^	4.30 ± 0.42 ^a^	6.68 ± 0.44^b^	2.33 ± 0.30 ^a^	3.53 ± 0.33 ^ab^	5.91 ± 0.36 ^b^
16	1	19.21 ± 0.28 ^ab^	n.d.	n.d.	n.d.	n.d.	n.d.	2.65 ± 1.00 ^ab^	n.d.	n.d.	9.08 ± 1.34 ^ab^	n.d.	n.d.	n.d.	n.d.	3.57 ± 0.71 ^ab^
16	4	45.42 ± 3.84 ^c^	n.d.	3.17 ± 1.30 ^b^	1.78 ± 0.65 ^bc^	0.94 ± 0.41 ^ab^	7.84 ± 1.51 ^c^	2.93 ± 0.59 ^ab^	5.98 ± 2.36 ^bc^	5.31 ± 2.30 ^a^	16.75 ± 0.80 ^cde^	4.57 ± 1.48 ^a^	3.10 ± 0.75^a^	1.64 ± 0.97 ^a^	6.32 ± 0.99 ^cd^	4.36 ± 0.56 ^ab^
16	7	66.45 ± 5.29 ^d^	5.17 ± 0.38 ^a^	3.42 ± 0.79 ^b^	7.70 ± 1.60 ^e^	4.18 ± 1.36 ^c^	15.39 ± 0.01 ^d^	4.83 ± 0.59 ^c^	18.43 ± 1.88 ^e^	26.18 ± 3.50 ^b^	25.25 ± 4.35 ^f^	12.07 ± 0.64 ^b^	6.75 ± 1.71^b^	7.19 ± 2.02 ^b^	7.87 ± 0.41 ^d^	6.40 ± 4.44 ^b^
20	2	36.56 ± 3.55 ^c^	n.d.	4.24 ± 1.49 ^b^	1.03 ± 0.42 ^ab^	n.d.	2.09 ± 0.06 ^a^	3.29 ± 0.02 ^b^	4.37 ± 0.84 ^b^	3.21 ± 0.99 ^a^	10.39 ± 2.13 ^b^	2.43 ± 0.51 ^a^	1.88 ± 0.39^a^	1.17 ± 0.33 ^a^	1.87 ± 0.16 ^a^	4.19 ± 0.87 ^ab^
20	6	63.15 ± 4.04 ^d^	6.43 ± 1.99 ^a^	11.50 ± 1.37 ^d^	3.41 ± 0.56 ^cd^	n.d.	17.03 ± 1.41 ^d^	4.77 ± 0.16 ^c^	12.18 ± 0.99 ^d^	6.37 ± 1.43 ^a^	18.60 ± 3.48 ^de^	11.20 ± 0.49 ^b^	2.34 ± 0.10^a^	8.10 ± 1.45 ^b^	4.65 ± 1.37 ^bc^	7.45 ± 0.38 ^b^
21	4	62.68 ± 7.78 ^d^	8.28 ± 1.25 ^a^	5.02 ± 1.20 ^bc^	3.70 ± 0.89 ^d^	n.d.	25.61 ± 0.59 ^e^	5.40 ± 1.35 ^c^	25.88 ± 0.41 ^f^	29.64 ± 0.32 ^b^	19.05 ± 3.08 ^e^	9.83 ± 3.49 ^b^	6.17 ± 0.49 ^b^	3.71 ± 0.39^a^	10.84 ± 1.31 ^e^	4.07 ± 2.08 ^ab^
**MANOVA**																
T	MS	896.34 ***	89.19 ***	45.83 ***	6.11 ***	1.88 **	411.11 ***	8.35 **	431.37 ***	598.74 ***	17.14	71.26 ***	12.47 **	28.38 ***	44.57 ***	10.22 ***
t	MS	2244.89 ***	37.55 ***	73.04 ***	50.43 ***	13.49 ***	294.41 ***	8.57 **	320.48 ***	537.88 ***	205.62 ***	155.90 ***	47.44 ***	66.41 ***	89.69 ***	47.45 ***

Data are mean values (expressed as mg GAE/100 g dw for TSPC and mg/100 g dw for individual compounds) ± standard deviation of three replicates. Different letters in the same column indicate significant differences for mean values (one-way ANOVA, post hoc Duncan´s test, *p* ≤ 0.05). Significance indicators: ** *p* ≤ 0.01 and *** *p* ≤ 0.001 (MANOVA). Abbreviations: 1-acetoxypinoresinol (1-AP); apigenin-6-*C*-arabinoside-8-*C*-hexoside (AAH); apigenin-6/8-*C*-pentoside-8/6-*C*-hexoside (APH); apigenin-6-*C*-galactosyl-8-*C*-glucosyl-*O*-glucuropyranoside (AGGG); caffeic acid *O*-hesoxide (CAH); dihydroferulic acid (DFA); *trans*-ferulic acid (FA); feruloyl quinic acid (FQA); isomer (i); isovitexin (IVT); mean of squares (MS); methyl isoorientin-2-*O*-rhamnoside (MIR); not detected (n.d.); temperature (T); time (t); total soluble phenolic compounds (TSPC); vicenin-2 (V-2); vitexin (VT).

**Table 2:** Antioxidant and anti-inflammatory activities of whole-wheat grain (control) and sprouts obtained at different temperatures and times.

T (°C)	t (d)	ORAC (μmol TE/100 g dw)	ABTS (μmol TE/100 g dw)	FRAP (mmol Fe^2+^/g dw)	DPPH (μmol TE/100 g dw)	TNF-α (% Inhibition)	IL-6 (% Inhibition)
Control		889.46 ± 146.87 ^a^	1061.01 ± 325.48 ^a^	0.11 ± 0.02 ^a^	217.725 ± 6.31 ^bc^	60.52 ± 4.23 ^a^	63.73 ± 8.97 ^cd^
12	2	854.29 ± 78.20 ^a^	716.02 ± 40.20 ^a^	0.12 ± 0.01 ^a^	135.77 ± 9.47 ^a^	67.63 ± 0.58 ^a^	42.94 ± 2.34 ^a^
12	6	1377.03 ± 188.52 ^b^	2089.69 ± 288.95 ^b^	0.14 ± 0.00 ^b^	304.65 ± 23.77 ^c^	67.63 ± 1.73 ^b^	66.87 ± 6.24 ^cd^
16	1	499.97 ± 53.06 ^a^	545.30 ± 66.15 ^a^	0.12 ± 0.00 ^a^	145.42 ± 29.44 ^ab^	63.12 ± 2.77 ^a^	70.05 ± 8.57 ^d^
16	4	1686.45 ± 77.95 ^b^	2285.16 ± 3.21 ^bc^	0.14 ± 0.01 ^bc^	251.44 ± 35.14 ^c^	76.77 ± 1.40 ^c^	74.81 ± 2.69 ^d^
16	7	2352.85 ± 209.80 ^c^	2985.91 ± 71.68 ^d^	0.16 ± 0.02 ^d^	357.29 ± 35.88 ^d^	70.94 ± 1.85 ^b^	47.18 ± 7.45 ^ab^
20	2	1370.32 ± 23.49 ^b^	1824.53 ± 369.00 ^b^	0.13 ± 0.00 ^ab^	259.33 ± 53.17 ^c^	69.60 ± 1.62 ^b^	56.57 ± 6.12 ^bc^
20	6	2058.28 ± 171.74 ^c^	2872.90 ± 319.94 ^cd^	0.15 ± 0.01 ^cd^	320.73 ± 16.16 ^d^	70.44 ± 1.54 ^b^	47.38 ± 2.68 ^ab^
21	4	2291.33 ± 105.87 ^c^	2862.50 ± 392.85 ^cd^	0.16 ± 0.01 ^d^	371.75 ± 38.63 ^d^	70.48 ± 1.75 ^b^	69.51 ± 12.28 ^d^
**MANOVA**							
T	MS	1082628 ***	2122616 ***	0.0004 **	42698.12 ***	105.90 ***	47.39
t	MS	2837502 ***	6166451 ***	0.0015 ***	60195.06 ***	144.19 ***	650.48 ***

Data are mean values ± standard deviation of three replications. Different letters in the same column indicate significant differences for mean values (one-way ANOVA, post hoc Duncan´s test, *p* ≤ 0.05). Significance indicators: * *p* ≤ 0.05, ** *p* ≤ 0.01 and *** *p* ≤ 0.001 (MANOVA). Abbreviations: Trolox equivalents (TE); 2,2’-azino-bis(3-ethylbenzothiazoline-6-sulfonic acid) (ABTS); 2,2-diphenyl-1-picryl-hydrazyl-hydrate (DPPH); ferric reducing antioxidant power (FRAP); interleukin (IL); mean of squares (MS); oxygen radical absorbance capacity (ORAC); temperature (T); time (t); tumor necrosis factor (TNF).

**Table 3:** Analysis of variance and regression coefficients of the predicted models for the response variables of germinated wheat.

Response Variable	Mathematical Models *	R^2^	R^2^-Adj
TPSC	Y=−27.10+2.65T+6.96t	0.909	0.903
1-AP	$Y=28.73-3.45T+0.10T_{}^{2}-3.63t+0.15t_{}^{2}+0.20\mathrm{Tt}$	0.816	0.782
APH (i1)	$Y=-2.67+0.22T-0.65t+0.20t_{}^{2}$	0.779	0.756
AGGG	$Y=15.69-3.14T+0.105T_{}^{2}+0.88t-0.39t_{}^{2}+0.30{\mathrm{Tt}}_{}$	0.807	0.771
CAH	Y=−2.38+0.28T+0.33t	0.760	0.743
FQA	Y=0.14Tt	0.915	0.906
MIR	$Y=-2.82t+0.21t_{}^{2}+0.14\mathrm{Tt}$	0.946	0.938
VT	$Y=-12.69+1.66T-0.04T_{}^{2}+0.25t_{}^{2}-0.04\mathrm{Tt}$	0.943	0.932
ORAC	$Y=-1279.86+89.57T+516.06t-35.17t_{}^{2}$	0.858	0.843
ABTS	$Y=-2093.80+123.80T+784.74t-53.37t_{}^{2}$	0.936	0.930
DPPH	Y=−164.04+16.72T+36.39t	0.765	0.750
TNF-α	$Y=-57.13+12.67T-0.35T_{}^{2}+12.44t-1.04t_{}^{2}-0.18\mathrm{Tt}$	0.891	0.870

* Models significance *p* ≤ 0.05 and lack of fit *p* ≥ 0.05 (ANOVA). Abbreviations: 2’-azino-bis(3-ethylbenzothiazoline-6-sulfonic acid) (ABTS); 1-acetoxypinoresinol (1-AP); apigenin-6/8-*C*-pentoside-8/6-*C*-hexoside (APH); apigenin-6-*C*-galactosyl-8-*C*-glucosyl-*O*-glucuropyranoside (AGGG); caffeic acid *O*-hexoside (CAH); 2,2-diphenyl-1-picryl-hydrazyl-hydrate (DPPH); feruloylquinic acid (FQA); isomer (i); methyl isoorientin-2-*O*-rhamnoside (MIR); oxygen radical absorbance capacity (ORAC); total soluble phenolic compounds (TSPC); tumor necrosis factor (TNF); temperature (T); time (t); vitexin (VT).

**Table 4:** Combination of germination factors and predicted values at optimum desirability (D) for phenolic content, antioxidant and anti-inflammatory activity.

Optimum *D* Value	Factors at Optimum *D* Value	Parameter	Predicted at Optimum *D*	Model Validation	
				Predicted at 20 °C, 6 d	Experimental at 20 °C, 6 d
0.8912	21 °C, 7 d	TSPC	74.44 ± 4.54	64.68 ± 2.72	63.15 ± 4.04
		1-AP	7.87 ± 0.62	5.88 ± 0.44	6.43 ± 1.99
		APH (i1)	7.26 ± 0.64	4.70 ± 0.38	11.50 ± 1.37
		AGGG	20.66 ± 0.71	18.91 ± 0.43	17.03 ± 1.41
		CAH	5.93 ± 0.45	4.99 ± 0.26	4.77 ± 0.16
		FQA	14.51 ± 1.20	10.84 ± 0.72	11.20 ± 0.49
		MIR	9.38 ± 0.81	6.24 ± 0.485	8.10 ± 1.45
		VT	10.56 ± 0.70	7.47 ± 0.41	7.45 ± 0.38
		ORAC	2490.26 ± 162.69	2244.39 ± 97.44	2058.28 ± 171.74
		ABTS	3384.37 ± 214.02	3041.39 ± 128.18	2872.90 ± 319.44
		DPPH	430.92 ± 59.13	352.27 ± 35.42	320.73 ± 16.16
		TNF-α	71.67 ± 1.85	71.72 ± 1.11	70.44 ± 1.54

Abbreviations: 2,2’-azino-bis(3-ethylbenzothiazoline-6-sulfonic acid) (ABTS); 1-acetoxypinoresinol (1-AP); apigenin-6/8-*C*-pentoside-8/6-*C*-hexoside (APH); apigenin-6-*C*-galactosyl-8-*C*-glucosyl-*O*-glucuropyranoside (AGGG); caffeic acid *O*-hexoside (CAH); 2,2-diphenyl-1-picryl-hydrazyl-hydrate (DPPH); feruloylquinic acid (FQA); isomer (i); methyl isoorientin-2-*O*-rhamnoside (MIR); oxygen radical absorbance capacity (ORAC); total soluble phenolic compounds (TSPC); tumor necrosis factor (TNF); vitexin (VT). Statistical errors estimated by the RSM model are calculated from replicated experimental data and include effects such as measurement error on the response, other sources of variation that are inherent in the system and effects of non-studied variables.

**Table 5:** Pearson’s correlation matrix of parameters across different experimental groups of wheat.

	ORAC	ABTS	FRAP	DPPH	TNF-α Inhibition	IL-6 Inhibition
TSPC	0.389 *	0.971 ***	0.866 ***	0.835 ***	0.6327 ***	−0.097
1-AP	0.600 **	0.717 ***	0.696 ***	0.719 ***	0.2596	−0.185
AAH	0.102	0.719 ***	0.505 *	0.626 ***	0.422 *	−0.102
APH (i1)	0.383 *	0.806 ***	0.822 ***	0.722 ***	0.4503 **	−0.229
AGGG	0.517 **	0.829 ***	0.815 ***	0.817 ***	0.500 **	0.026
CAH	0.544 ***	0.796 ***	0.767 ***	0.779 ***	0.423 *	−0.214
DFA (i1)	0.545 **	0.831 ***	0.817 ***	0.802 ***	0.448 **	0.014
DFA (i2)	0.557 **	0.704 ***	0.772 ***	0.713 ***	0.379 *	−0.008
FA	0.352 *	0.805 ***	0.679 ***	0.615 ***	0.485 **	−0.203
FQA	0.459 **	0.918 ***	0.826 ***	0.819 ***	0.555 ***	−0.155
IVT	0.217	0.741 ***	0.737 ***	0.677 ***	0.456 **	0.122
MIR	0.378 *	0.798 ***	0.697 ***	0.741 ***	0.426 *	−0.342
V-2	0.420 *	0.838 ***	0.806 ***	0.765 ***	0.6756 ***	0.225
VT	0.311	0.756 ***	0.724 ***	0.723 ***	0.466 **	0.027

Data represent the determination coefficients (*r*). Significance indicators: * *p* ≤ 0.05, ** *p* ≤ 0.01 and *** *p* ≤ 0.001. Abbreviations: 2,2’-azino-bis(3-ethylbenzothiazoline-6-sulfonic acid) assay (ABTS); 1-acetoxypinoresinol (1-AP); apigenin-6-*C*-arabinoside-8-*C*-hexoside (AAH); apigenin-6/8-*C*-pentoside-8/6-*C*-hexoside (APH); apigenin-6-*C*-galactosyl-8-*C*-glucosyl-*O*-glucuropyranoside (AGGG); caffeic acid *O*-hexoside (CAH); dihydroferulic acid (DFA); 2,2-diphenyl-1-picryl-hydrazyl-hydrate assay (DPPH); ferric reducing antioxidant power (FRAP); *trans*-ferulic acid (FA); feruloylquinic acid (FQA); interleukin (IL); isomer (i); isovitexin (IVT); methyl isoorientin-2-*O*-rhamnoside (MIR); oxygen radical absorbance capacity (ORAC); total soluble phenolic compounds (TSPC); tumor necrosis factor (TNF); vicenin-2 (V-2); vitexin (VT).

## Supplementary Materials

The following are available online at https://www.mdpi.com/2076-3921/9/5/426/s1, Figure S1: HPLC chromatogram of sprouted wheat phenolic profile extracted at 290 nm. Proposed phenolic compounds were numbered by elution order. Figure S2: Bidimensional contour plot for desirability (D) as function of germination temperature and time. Table S1: Chromatographic and mass spectrum data of the phenolic compounds identified in whole wheat grains and sprouts.
