# Supplementary material for: Soluble Phenolic Composition Tailored by Germination Conditions Accompany Antioxidant and Anti-Inflammatory Properties of Wheat
Source: Antioxidants (Basel). 2020 May 14;9(5):426. doi: 10.3390/antiox9050426 (PMC7278661; doi:10.3390/antiox9050426)
Supplement: Supplementary file 1 [file antioxidants-09-00426-s001.pdf]

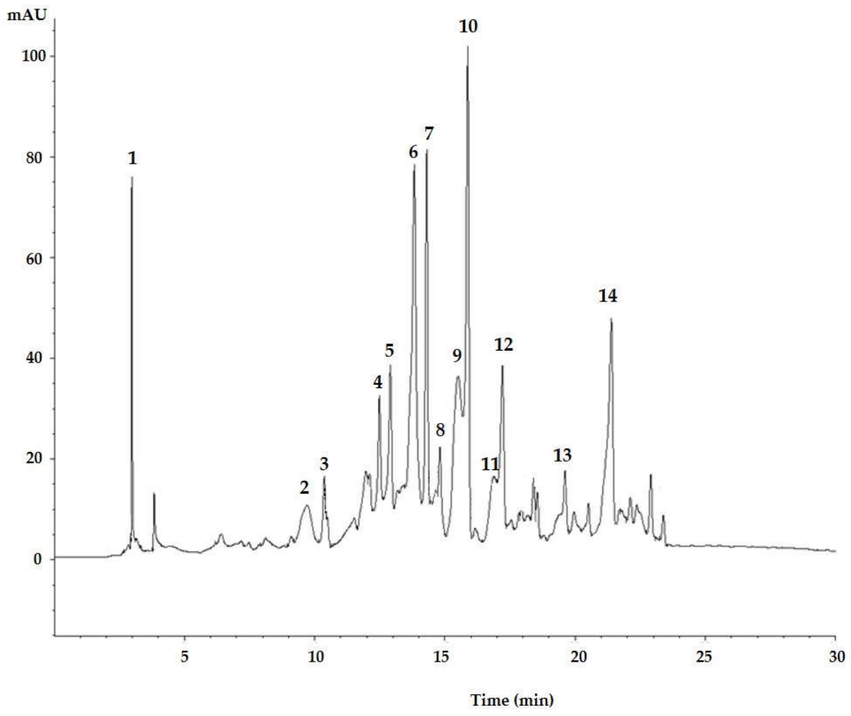

Figure S1. HPLC chromatogram of sprouted wheat phenolic profile extracted at 290 nm. Proposed phenolic compounds were numbered by elution order (See Table S1 for peak numbers).

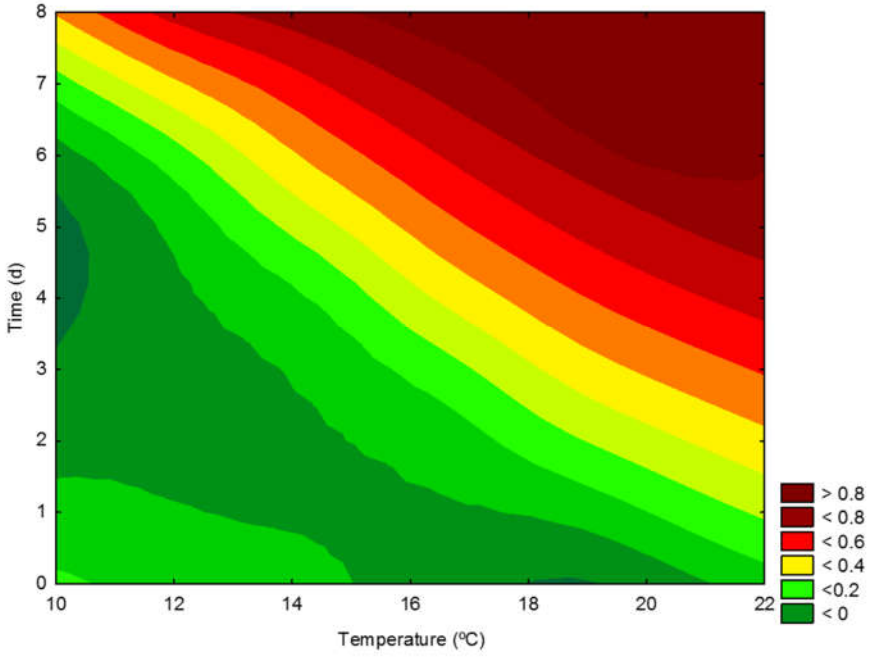

Figure S2. Bidimensional contour plot for desirability (*D*) as function of germination temperature and time.

Table S1. Chromatographic and mass spectrum data of the phenolic compounds identified in whole wheat grains and sprouts

Peak no.	[M-H]⁻ (*m*/*z*)	MS² (*m*/*z*)	RT (min)	Tentative identification	Phenolic class	Standard for quantification
1	355	179	2.9	Caffeic acid *O*-hexoside	Phenolic acid	Caffeic acid[1]
2	625	305	9.8	Apigenin-6/8-*C*-pentoside-8/6-*C*-hexoside (i1)	Flavone-*C*-glycoside	Vicenin-2[2]
3	625	305	10.5	Apigenin-6/8-*C*-pentoside-8/6-*C*-hexoside (i2)	Flavone-*C*-glycoside	Vicenin-2
4	607	323	12.2	Methyl isoorientin-2-*O*-rhamnoside	Flavone-*C*-glycoside	Vicenin-2
5	431	186	13	Vitexin (aginenin 8-*C*-glucoside)	Flavone-*C*-glycoside	Vicenin-2
6	367	193	13.9	Feruloylquinic acid	Phenolic acid	Ferulic acid[3]
7	564	269	14.3	Apigenin-6-C-arabinoside-8-*C*-hexoside	Flavone-*C*-glycoside	Vicenin-2
8	385	192	14.8	Dihydroferulic acid (i1)	Phenolic acid	Ferulic acid
9	385	192	15.5	Dihidroferulic acid (i2)	Phenolic acid	Ferulic acid
10	193	-	15.8	Ferulic acid	Phenolic acid	Ferulic acid
11	415	191	16.9	1-Acetoxypinoresinol	Lignan	Podophyllotoxin[4]
12	594	179	17.2	Vicenin-2 (apigenin-6,8-di-*C*-glucoside)	Flavone-*C*-glycoside	Vicenin-2
13	431	186	19.6	Isovitexin (aginenin 6-C-glucoside)	Flavone-*C*-glycoside	Vicenin-2
14	769	269	21.4	Apigenin-6-*C*-galactosyl-8-*C*-glucosyl-*O*-glucuropyranoside	Flavone-*C*-glycoside	Vicenin-2

RT: retention time; i: isomer;

[1] Linearity range 1-100 µg/mL, $r^2 > 0.99$

[2] Linearity range 1-50 µg/mL, $r^2 > 0.99$

[3] Linearity range 1-100 µg/mL, $r^2 > 0.99$

[4] Linearity range 1-50 µg/mL, $r^2 > 0.99$
